# Forebrain NR2B Overexpression Facilitating the Prefrontal Cortex Long-Term Potentiation and Enhancing Working Memory Function in Mice

**DOI:** 10.1371/journal.pone.0020312

**Published:** 2011-05-31

**Authors:** Yihui Cui, Jing Jin, Xuliang Zhang, Hao Xu, Liguo Yang, Dan Du, Qingwen Zeng, Joe Z. Tsien, Huiting Yu, Xiaohua Cao

**Affiliations:** 1 Shanghai Institute of Brain Functional Genomics, The Key Laboratories of MOE and STCSM, East China Normal University, Shanghai, China; 2 Brain and Behavior Discovery Institute and Department of Neurology, School of Medicine, Medical College of Georgia, Augusta, Georgia, United States of America; 3 Department of Vital Statistics, Shanghai Municipal Center For Disease Control and Prevention, Shanghai, China; University of Chicago, United States of America

## Abstract

Prefrontal cortex plays an important role in working memory, attention regulation and behavioral inhibition. Its functions are associated with NMDA receptors. However, there is little information regarding the roles of NMDA receptor NR2B subunit in prefrontal cortical synaptic plasticity and prefrontal cortex-related working memory. Whether the up-regulation of NR2B subunit influences prefrontal cortical synaptic plasticity and working memory is not yet clear. In the present study, we measured prefrontal cortical synaptic plasticity and working memory function in NR2B overexpressing transgenic mice. In vitro electrophysiological data showed that overexpression of NR2B specifically in the forebrain region resulted in enhancement of prefrontal cortical long-term potentiation (LTP) but did not alter long-term depression (LTD). The enhanced LTP was completely abolished by a NR2B subunit selective antagonist, Ro25-6981, indicating that overexpression of NR2B subunit is responsible for enhanced LTP. In addition, NR2B transgenic mice exhibited better performance in a set of working memory paradigms including delay no-match-to-place T-maze, working memory version of water maze and odor span task. Our study provides evidence that NR2B subunit of NMDA receptor in prefrontal cortex is critical for prefrontal cortex LTP and prefrontal cortex-related working memory.

## Introduction

Previous studies have demonstrated that the prefrontal cortex (PFC) plays an important role in working memory [1], [2], [3], emotional memory [4], attention regulation [5], [6], [7], and behavioral inhibition [8], [9]. In addition, it has been shown that NMDA receptor is crucial for the function of prefrontal cortex [10]. For example, antagonists of NMDA receptor impaired prefrontal cortex-dependent working memory [11]. The NMDA receptors are heteromeric complexes consisting of NR1 subunit, various NR2 subunits (A, B, C, D), and NR3 subunits (A, B) [12], [13], [14]. The formation of functional NMDA receptors requires a combination of NR1 and at least one of NR2 subunits. Among the four subunits, NR2A and NR2B subunits are predominantly expressed in adult forebrain regions including the hippocampus and cortex [15].

Although the roles of NR2A and NR2B subunits in hippocampal synaptic plasticity have been extensively investigated, their roles in the prefrontal cortical plasticity are not well characterized. So far, only Zhao MG et al [16] reported that NR2A or NR2B subunit antagonists blocked LTD and LTP in prefrontal cortex, indicating that the down-regulation of NR2B subunit function led to an attenuation of NMDAR- mediated LTP and LTD in prefrontal cortex. It has not been clear the influence of up-regulation of NR2B subunits on the prefrontal cortex synaptic plasticity and working memory function. In the present study, we used NR2B transgenic mice, in which NR2B subunits were overexpressed throughout the forebrain without alteration in expression level of NR2A subunits [17], and investigated effects of NR2B subunit overexpression on prefrontal cortex synaptic plasticity and working memory.

## Materials and Methods

### Ethics Statement

All mouse work described in this study have been conducted according to Animals Act, 2006 (China) and approved by the Institutional Animal Care and Use Committee (IACUC approval ID #M07016) of the East China Normal University.

### Synaptosomal Preparations and Immunoblot Analysis

Prefrontal cortex were dissected from adult (3-month old) NR2B transgenic mice and wild-type littermates, and synaptosomes were prepared essentially as described previously [18]. Briefly, brain tissues were homogenized in ice-cold HEPES-buffered sucrose (0.32 M sucrose, 5 mM HEPES, pH 7.4) containing freshly added protease inhibitor, and centrifuged at 1000× g for 5 min to remove the pelleted nuclear fraction. The supernatant was then centrifuged at 12,000× g for 20 min to yield the membrane fraction pellet. The pellet was then resuspended, loaded onto a discontinuous sucrose gradient (0.8, 1.0, and 1.2 M) and centrifuged at 85.000× g for 2 h. The synaptosomal fraction (all other proteins) was collected from the interface between the 1.0 and 1.2 M sucrose layers, then Krebs' solution(145 mM NaCl, 5 mM KCl, 1.2 mM MgCl_2_, 1.2 mM NaH_2_PO_4_, 10 mM Glucose, 20 mM HEPES(Na+),1.2 mM CaCl_2_) was added into the synaptosomal fraction and centrifuged at 12.000× g for 20 min. The pellet was resuspended in Synaptome Lysis buffer(25 mM HEPES, 10 mM MgCl_2_, 0.5 mM DTT, 0.5 mM EGTA, 1 mM EDTA, 1 µM PMSF,10 µM cocktail). Proteins were separated by electrophoresis on SDS-PAGE, and Western blots were performed according to standard protocols. The following primary antibodies were used at the concentrations given: NR2B at 1∶1000 (Upstate), β-actin at 1∶1000 (Cell signaling) and HRP-conjugated secondary goat anti-rabbit (Upstate) at 1∶20000. Blots were developed using ECL chemiluminescence substrate (Pierce) onto x-ray films (Kodak). Bands were quantified using Quantity One Software (Bio-Rad). The results were shown as mean ± SEM and statistically analyzed using Student's *t*-test,

### Electrophysiological Recording of Prefrontal Cortex Slice

The coronal sections that contain prefrontal cortex formations were prepared according to the method as described previously [16]. Briefly, mice were anaesthetized with sodium pentobarbital and were sacrificed by decapitation. Transverse slices of the prefrontal cortex (380 µm) were cut using the vibratome in the ice-cold modified artificial cerebrospinal fluid (mACSF) consisting of 110 mM Choline chloride, 2.5 mM KCl, 0.5 mM CaCl_2_, 7 mM MgSO_4_, 25 mM NaHCO_3_, 1.25 mM NaH_2_PO_4_, 25 mM D-glucose, and 3.1 mM Na pyruvate, which is saturated with 95% O_2_ and 5% CO_2_. Slices were transferred to a incubating chamber with oxygenated (95% O_2_ and 5% CO_2_) normal ACSF containing 120 mM NaCl, 2.5 mM KCl, 2.5 mM CaCl_2_, 1.3 mM MgSO_4_, 26 mM NaHCO_3_, 1 mM NaH_2_PO_4_, 10 mM D-glucose, pH 7.3–7.4, and incubated for 1 h at 30°C. During recording, a bipolar tungsten stimulating electrode was place in the layer V of prefrontal cortex. We recorded the extracellular field excitatory postsynaptic potential (fEPSPs) from the layer II–III neurons of prefrontal cortex using glass microelectrode (4–8 MΩ, filled with 0.5 M natrium aceticum). Test responses were elicited at 0.033 Hz. After recording a stable baseline for at least 15 min, LTP was induced by high-frequency stimulation (100 Hz for 1 s, 2 trains, 30 s interval). Data were presented as the mean ± SEM. Student's t-test and Tukey's HSD post-hoc test was used for statistical analysis.

### T-maze Task

The protocol is the same as described previously [19]. The T-maze consists of a start arm (length 57 cm, width 10 cm and height 10 cm) and two identical goal arms (length 40 cm, width 10 cm and height 10 cm). There was a food well located 3 cm from the end of each goal arm. Before the training sessions, mice were housed individually and maintained on a restricted feeding schedule at approximately 85% of their pre-experimental body weight. Then, mice were habituated to the maze and were accustomed to reward food (small sugar pellet). Each trial consisted of a force-run and a choice-run. For the force-run, the mouse was forced to enter either left or right arm to get the food (a small sugar pellet) by blocking a door. The direction of the forced run was random but no more than 2 times allowed in the same direction consecutively. For the choice-run, the blocked door was removed and the mouse was allowed to choose either arm freely. When the mouse entered the previously unvisited arm, the reward was given. The interval between the force-run and the choice-run was 15 s. The training session lasted until the correct performance was stabilized at 85% for two consecutive days. During retention session, the interval between the force–run and the choice run was prolonged to 1 and 3 min. Between each run, the arms were cleaned with 75% alcohol to remove the effect of olfactory quickly. Each block consisted of a total of eight trials, conducted in two consecutive days with four trials per day. Behavioral performance was analyzed by a two-way repeated measures ANOVA and Student's t-test.

### Modified Water Maze Task

The water maze consists of circular pool, 150 cm diameter and 50 cm height, filled with the white opaque water (22±0.5°C). This experimental procedure includes pre-training and training. During pre-training, the visible platform was located in a fixed position in the center of pool throughout four trials. For each trial, the mice was gently released into the pool. The placement location was at the edge of the pool, facing the wall, in the randomized quadrant. The mouse was required to find the platform within 60 s. If failed, it was guided to the platform by the experimenter. The mouse was allowed to remain on the platform for 20 s. Latency to reach the visible platform is measured. Swim speed is calculated. After pre-training, training on the working memory version of water maze task started. Mice were trained two trials per day for 4 consecutive days. The hidden platform was placed at the different position of pool every day but the same position across two trials on the same day. The points of releasing mice were different but distance to the invisible platform position was constant. The time interval between the first and second trial was approximately 30 s. The escape latency and swimming length to the invisible platform were automatically recorded by Track Video Analysis System (Coulbourn instrument, USA). This task assessed the mice' ability to use spatial cues from the first trial of each day to enhance performance on the second trial. Thus, Improvement of latency between trial 1 and 2 reflects working memory. The behavioral performances were analyzed by two-way repeated measures ANOVA and Tukey's HSD post-hoc test was used to analyze the difference between groups at each trail.

### Odor Span Task

The procedure is similar to the protocol described previously by Young et al [20]. Briefly, 20 different odors (Elan Flavors & Fragrances CO., LTD), for which mice showed no preference, were used in this experiment. The different scented mixtures were prepared by mixing each odor with the woodchip (bedding material) respectively. The cereal reward pellets were buried in a porcelain cup (5.5 cm in diameter * 2.5 cm high) with the unscented or different scented woodchip. All used cups are same in texture, size and shape in this study. In addition, to exclude the complicated influence of mouse marking cup and woodchip, all used cups and woodchip were replaced with new cups and woodchip between trials or spans.

The experiment consists of shaping, odor non-matching to sample (NMS) task, odor span task and no reward probe. Before shaping, mice were individually handled and habituated to a gray box (50*50*25 cm high), the porcelain cup and the cereal reward pellets.

#### Shaping

On day 1, one unscented cup with 20 buried reward pellets was placed in the gray box, then a mouse was introduced to the box. The mouse was removed until the 20 buried reward pellets were dug out and consumed. On day 2, two unscented cups (one baited with 10 pellets) were put into the gray box, the mouse was then transferred to the box and required to dig and consume all the 10 pellets. This procedure was immediately repeated one more time.

#### Non-matching to sample (NMS) task

The mouse was trained to learn the ‘non-matching to sample’ rule for at least 4 days (10 trials per day). In trial 1, a random scented cup (e.g., cup A), containing the random one of 20 different odor mixtures and 2 buried reward pellets (termed cup A+), was placed in the box before introducing the mouse. After the mouse consumed 2 pellets, both the mouse and cup A were removed from the box. In trial 2, another cup (refilled the same scented mixture as cup A but not baited, termed cup A-), and a second new scented cup with 2 buried pellets (termed cup B+) were randomly distributed in box. Following consumption of the pellets, both mouse and two cups were removed. In trial 3, before mouse was positioned in the box, a third novel scented cup with 2 pellets (cup C+) and another cup B- were randomly positioned in the box. When the reward pellets were consumed, trial 4 started. This process was repeated 6 more times (total 10 trials). This rule training lasted for at least 4 more days until mouse dug up and consumed all the 20 pellets in less than 8 min.

#### Odor span task

**Figure 1:**
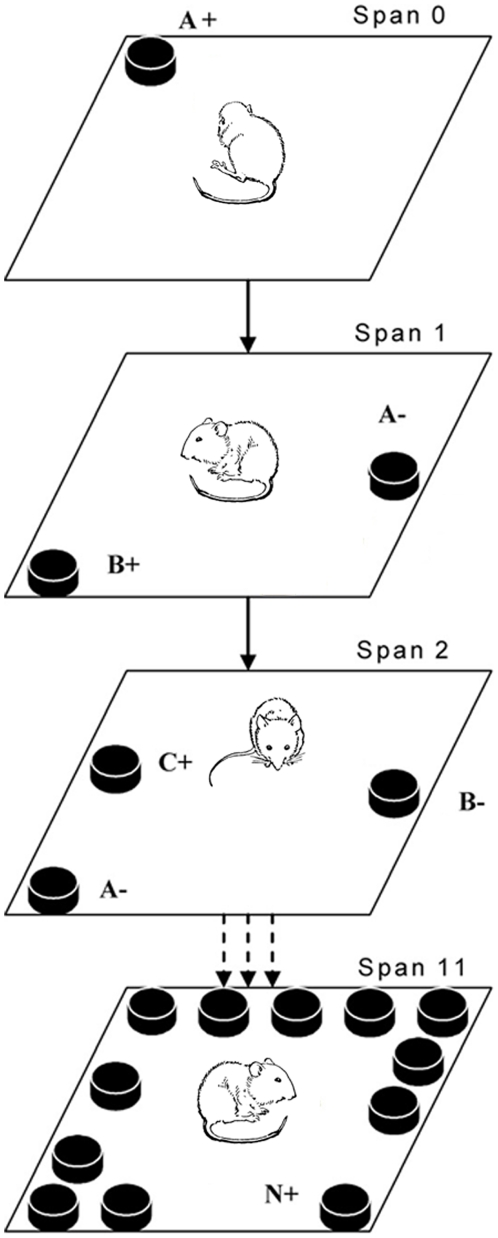
Diagram of the Odor Span Task. At span 0, mice are first presented with a random scented cup buried 2 pellets (e.g., A+). After consumption of the reward, the mouse and the cup were removed. At span 1, a second new scented cup with 2 pellets (e.g., B+) and another cup refilled with A odor woodchip without reward (e.g., A-) was pseudo-randomly relocated in the box. Mice were return to the box and were required to remember odor A and to dig at the cup with the new B odor. Then, additional cups of woodchip scented with different odors were placed in the same manner until 12 cups (span 11) were presented.

After learning ‘non-matching to sample’ rule, the mouse was subject to the odor span task (Figure 1). The correct response of mouse is to dig the novel scented cup, which is not presented to the mouse at previous spans of a session. The procedure is similar to that of NMS task. Briefly, at span 0, a random scented cup with 2 pellets (e.g., cup A+) was pseudo-randomly placed in the box. After consumption of the reward, the mouse and the cup was removed. At span 1, a second new scented cup with 2 pellets (e.g., cup B+) was randomly selected and the location was randomly generated, and another cup refilled with A odor woodchip without reward (cup A-) was pseudo-randomly relocated in the box. If the mouse dug in the novel scented cup (cup B+) and it was allowed to consume the reward, and span 2 started with a third novel scented cup (e.g., cup C+), the refilled cup A- and B- were placed at the randomly selected locations. If incorrect, the mouse was removed and the refilled cup B+ and cup A- were randomly relocated, span 1 was repeated until a correct choice was made. The accuracy of this span (2-odor discrimination) was recorded and was considered as a measure of the olfactory discriminatory ability. The span numbers increased with every correct response until span 11 was reached. As soon as mouse making incorrect response, mouse and all cups removed from the box, cups were refilled with same odor but previously non sampled scented mixture and relocated randomly to repeat the same span. If a mouse made 10 consecutive incorrect responses, the task would be ended. Between mice, the box was wiped down with ethanol (75%).

The number of correct choice prior to the first error was regarded as the span length of that mouse for that session. The total number of spans completed by each mouse, regardless of the number of incorrect responses, was recorded. Accuracy ([total number of completed spans/(total number of completed spans + total errors)]*100%) and mean span latency (total time/total number of completed spans) were also calculated. Acquisition criteria was defined as a span length when the performance of mice was significant greater than that of day 1 (session 1). After reaching acquisition criteria, training continued until two groups exhibited a stable level of performance with span length fluctuating within a maximum of 3 spans over 4 consecutive days. The performance of the two groups in the odor span task was compared using a two-way ANOVA. In addition, the effect of genotype on stable performance was assessed by comparing simple 2-odor discrimination, span length, % accuracy and total errors of two groups across 4 consecutive sessions with Mann-Whitney Rank Sum test. Mean span latency was compared using repeated-measures ANOVA.

#### No reward probe session

To test whether the scent of the buried reward pellets controlled behavior, the no reward probe session (session 17) was performed[21]. In this session, the mouse was presented with the increasing numbers of scented cups in the same way as the above session of odor span task. No cereal reward, however, was buried in the correct cups. The cereal pellets were dropped into the cup only after the mouse dug in the correct cup.

## Results

### Expression of NR2B Protein in NR2B Transgenic Mice

**Figure 2:**
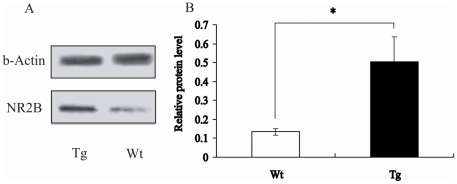
Synaptosome NR2B-receptor Protein in Prefrontal Cortex. Analysis of OD value show that the relative quantity of NR2B-receptor protein of prefrontal cortex in Tg and Wt mice is 0.5±0.1 and 0.13±0.02, respectively (p<0.05, Student's *t*-test).

Using western blot techniques, we first measured the NR2B protein of synaptosomal membrane fractions prepared from the prefrontal cortex of NR2B transgenic and wild type mice. Our western blot results showed that there was an enhanced expression of NR2B protein in the synaptic membrane of prefrontal cortex of transgenic mice compared to the wild type mice (Figure 2).

### Enhanced Prefrontal cortex LTP in Transgenic NR2B Mice

**Figure 3:**
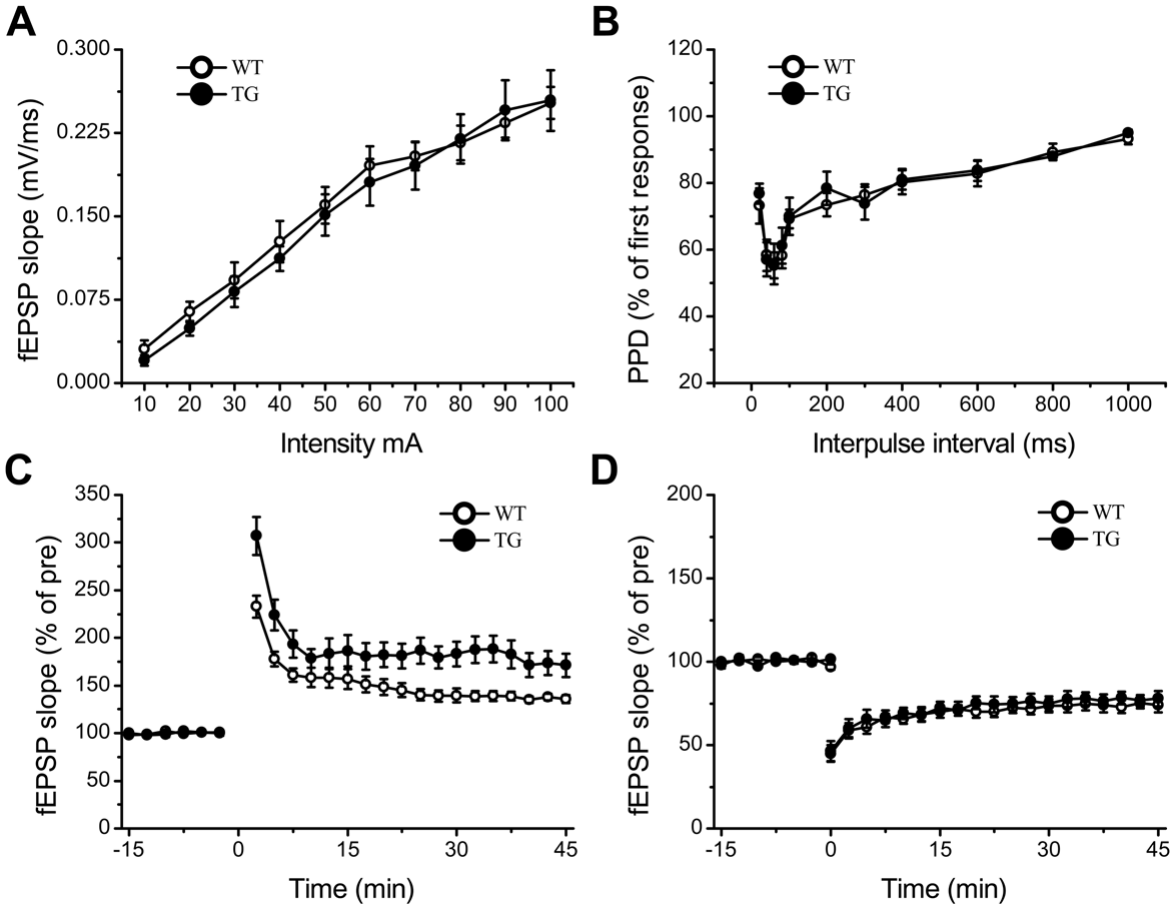
NR2B Overexpression Enhanced LTP but not Basal Transmission and LTD in prefrontal cortex. A: No significant difference in input-output curve between Tg and Wt slices. B: No significant difference in pair-pulse responses between Tg and Wt slices. C: LTP induced by tetanic stimulations in Tg slices were significantly larger than that of Wt slices. D: LTD induced by a low frequency stimulation in Tg slices were not significant different from that of Wt slices. All data are presented as mean ± SEM. Statistical differences were evaluated with student's *t* -test.

To examine effect of NR2B overexpression on the synaptic transmission of prefrontal cortex in the transgenic NR2B mice, we investigated the synaptic plasticity in prefrontal cortex of NR2B transgenic mice using *in vitro* field potential recording technique. As shown in Figure 3A and B, there was no significant difference in basal synaptic transmission and pair-pulse depression (PPD) between transgenic and wild-type slice, suggesting that the overexpression of the NR2B subunits does not change basic synaptic transmission and presynaptic function. However, the high frequency stimulation (100 Hz for 1 s, 2 trains, 30 s interval) evoked significantly larger LTP in Tg slices than in Wt slices (Figure 3C; Tg, 174.4±12.6%, n = 9 slices/7mice; Wt, 136.7±3.5%, n = 10 slices/7 mice; p<0.05 compared to Tg mice). In addition, NMDA receptor antagonist, 100 µM AP-5, completely blocked the enhanced LTP (data not shown), suggesting the enhanced LTP was NMDA receptor dependent. The prefrontal cortex LTD was also examined in Tg and Wt mice. No significant difference was measured in prefrontal cortex LTD between Wt (Figure 3D, 74.03±4.39%, n = 9slices/5 mice) and Tg mice (72.26±3.69%, n = 11 slices/4 mice t-test, p>0.05 vs Wt mice), suggesting that overexpression of NR2B subunit does not affect the induction of LTD at the prefrontal cortex.

### Contribution of NR2B Overexpression to the Enhanced LTP in Transgenic NR2B Mice

**Figure 4:**
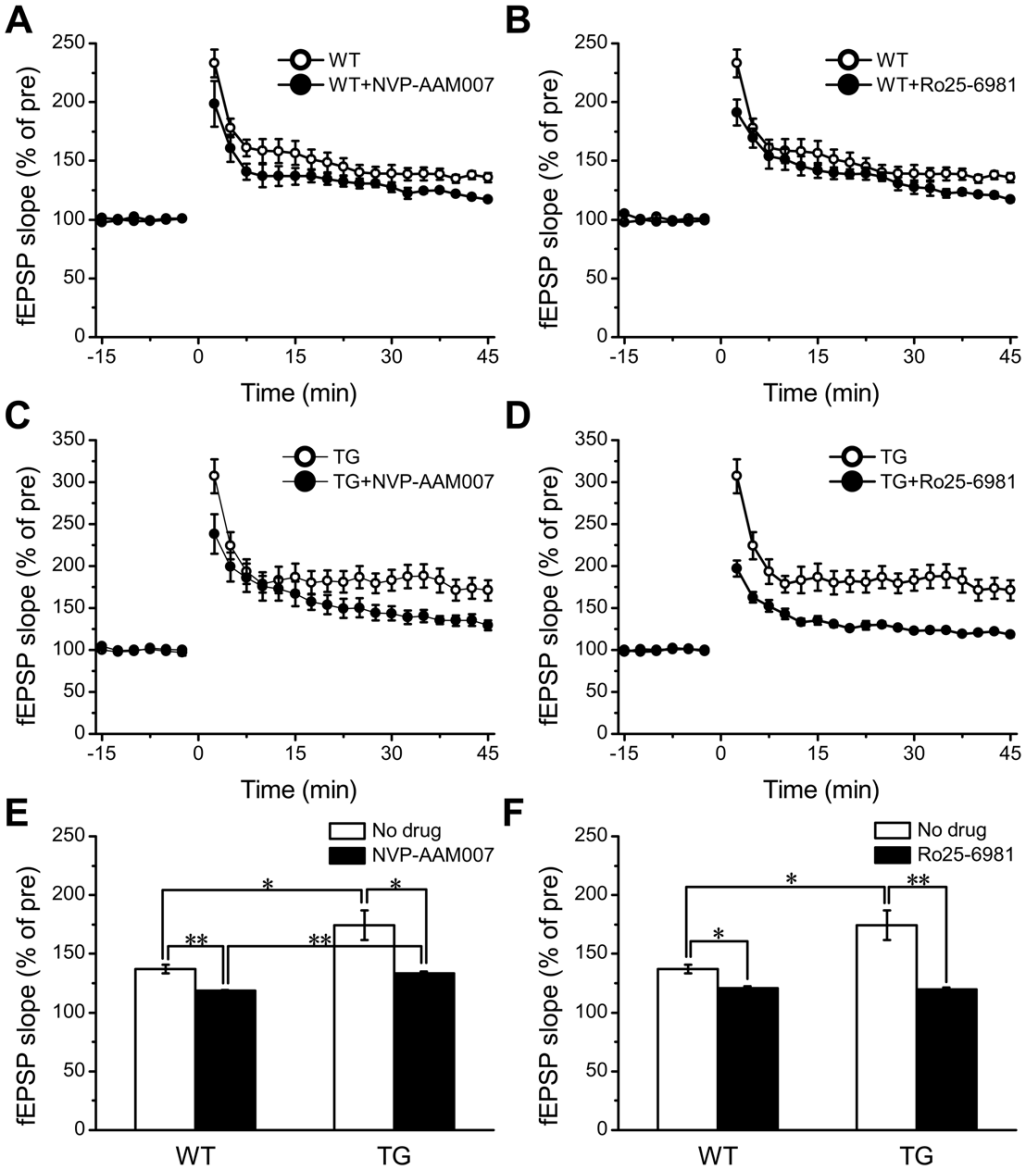
The Role of NR2B Subunit in Enhanced Prefrontal-LTP in Transgenic Slices. A: NR2A-selectice antagonist (NVP-AAM077) reduced prefrontal cortex l LTP in Wt slices. B: NR2B-selectice antagonist (Ro25-6981) also reduced prefrontal cortex LTP in Wt slices. C: Effect of NVP-AAM077 on prefrontal cortex LTP in Tg slices. D: Ro25-6981 had much larger effect on prefrontal cortex LTP in Tg slices. E: Statistical analysis shows the effects of NVP-AAM077 on prefrontal cortical LTP in both Tg and Wt slice, it indicates a significant involvement of NR2A subunits in prefrontal cortex LTP of both Tg and Wt slices. F: Statistical analysis shows the effects of Ro25-6981 on prefrontal cortex LTP in both Tg and Wt slice, suggesting a significant involvement of NR2B subunits in prefrontal cortex LTP of both Tg and Wt slices. All values are mean ± SEM. Statistical differences were evaluated with Student's *t* –test (A, B, C, D) and Tukey's HSD post-hoc test (E, F)(*denotes p<0.05, **denotes P<0.01).

To evaluate the contribution of NR2A and NR2B subunits to prefrontal cortex LTP, the selective antagonists of NMDA receptor subunits were applied to the prefrontal slices. NVP-AAM077 and Ro25-6981 are selective antagonists for NR2A-containing NMDARs and NR2B-containing NMDARs, respectively [16]. In Wt slices, LTP was significantly reduced but not completely blocked by 0.4 µM NVP-AAM077 (Figure 4A, 118.8±1.2%; n = 6 slices/2 mice, Student's *t*-test, p<0.01) or 0.3 µM Ro 25–6981 (Figure 4B, 120.6±1.9%; n = 8slices/3mice, Student's *t*-test, p<0.05), respectively. Similarly, NVP-AAM077 or Ro 25–6981 also reduced LTP in the transgenic slices (Figure 4C, Tg with NVP: 133.6±5.6%, n = 6 slices/2 mice, p<0.05; Figure 4D, Tg with Ro25: 119.7±1.4%, n = 7 slices/3 mice, p<0.01). These results suggest that both NR2B and NR2A subunits contribute to the induction of prefrontal LTP in both Wt and Tg slice. Especially, under the NVP-AAM077 treatment, LTP in Tg slices was significantly larger than that of Wt slices (Figure 4E, Tg: 133.6±5.6%; n = 6 slices/2 mice; Wt: 118.8±1.2%; n = 6 slices/2 mice, Tukey's HSD post-hock test, p<0.05). In addition, under the Ro 25–6981 treatment, LTP of Wt slices was comparable with that of Tg slices (Figure 4F, Wt: 120.6±1.9%, n = 8 slices/3mice; Tg: 119.7±1.4%, n = 7 slices/3 mice; Tukey's HSD post-hock test, p>0.05). These results suggest that overexpression of NR2B subunit is responsible for enhanced prefrontal cortex LTP in Tg slices.

### Enhanced Spatial Working Memory in Transgenic NR2B Mice

A number of studies demonstrate that PFC is crucial for working memory [22], [23], [24], and it also has been reported that the administration of NMDA receptor antagonists impairs spatial working memory in rats [25]. To investigate whether NR2B overexpression in forebrain can influence prefrontal cortex-related working memory, NR2B transgenic mice were tested on T-maze non-matching delayed alternation task, modified water maze task and odor span task.

**Figure 5:**
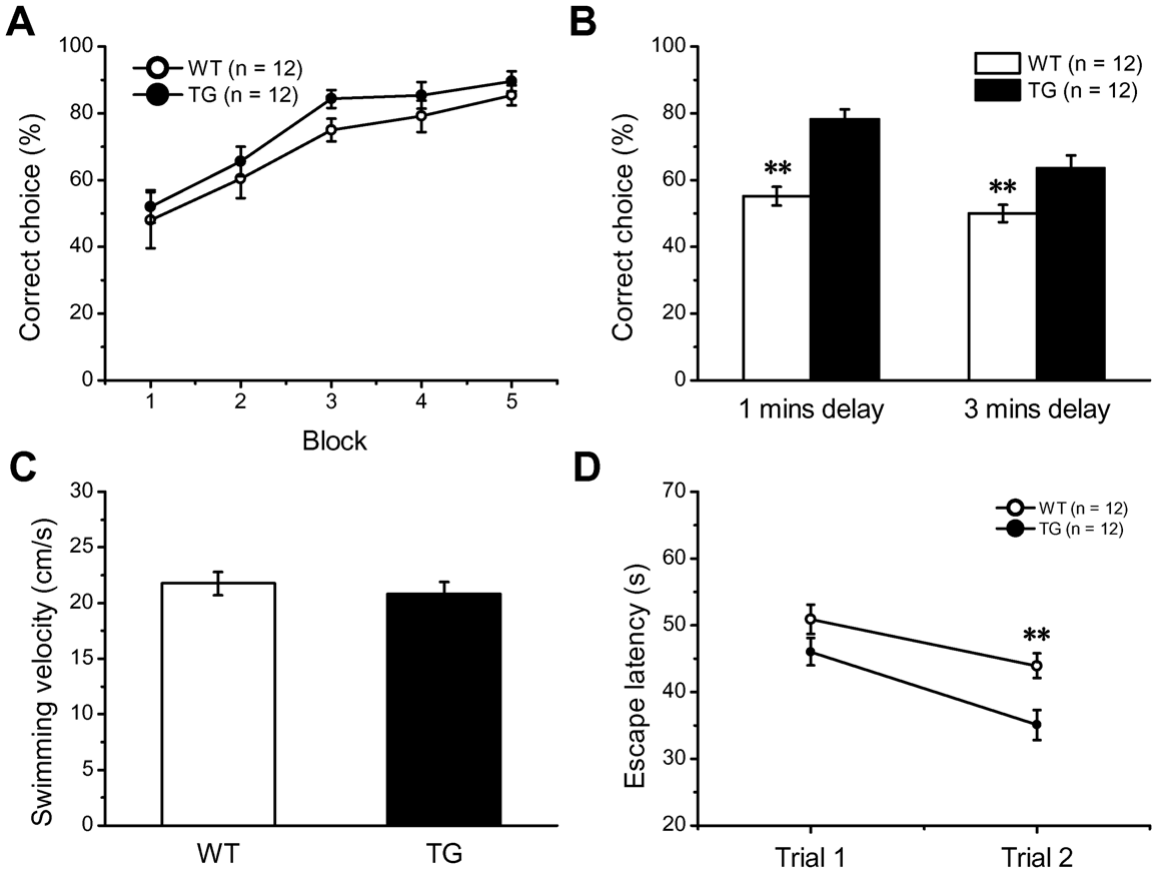
Enhancement of Spatial Working Memory in NR2B Transgenic Mice. A–B: Performance of mice in T-maze task. A: There was no difference in accuracy between Wt and Tg mice in training session. B: Tg mice exhibited superior performance both in 1- and 3 min-delay retention test. C–D: Performance of mice in the working memory version of water maze task. C: There was no difference in swim speed between Wt and Tg mice in pre-training. D: In the 2^nd^ trial of training, the latency of transgenic mice was significantly shorter than that of wild type. All values are mean ± SEM (**denotes p<0.01 when compared to Wt controls).

In the training session of T-maze task, the accuracy of NR2B transgenic mice and Wt mice was comparable (F_(1,22)_ = 1.75, p>0.05, two-way repeated measures ANOVA, Figure 5A). However, the accuracy of Wt mice was significantly reduced compared to that of Tg mice during 1 min (Wt: 55.2%; Tg: 75.1%, p<0.01 compared to Wt) and 3 min retention test (Wt: 50%, Tg: 63.5%; p<0.01 compared to Wt, Figure 5B). This result indicates that the transgenic mice have better spatial working memory.

To further confirm and extend the above result, spatial work memory of all mice were measured using a working memory version of water maze task. During pretraining, no significant difference was observed in swim speed between the transgenic mice and their wild type littermates (Figure 5C), suggesting NR2B overexpression did not impact the mice's motivation in escaping from the water and swimming ability.

During training of working memory version of water maze task, a two-way repeated measures ANOVA reveals significant effect of both trial (F_(1,22)_ = 14.80; p<0.01, Figure 5D) and group (F_(1,22)_ = 14.99; p<0.01, Figure 5D) on latency, but no group-by-trial interaction was observed (F_(1,22)_ = 0.94, p>0.05). Further analysis on latency using Tukey's HSD post-hoc test shows a significant difference in trial 2 (p<0.01) not in trial 1 (p>0.05) between two groups, suggesting spatial working memory in transgenic mice have been enhanced.

### Enhanced Non-spatial Working Memory in Transgenic NR2B Mice

**Figure 6:**
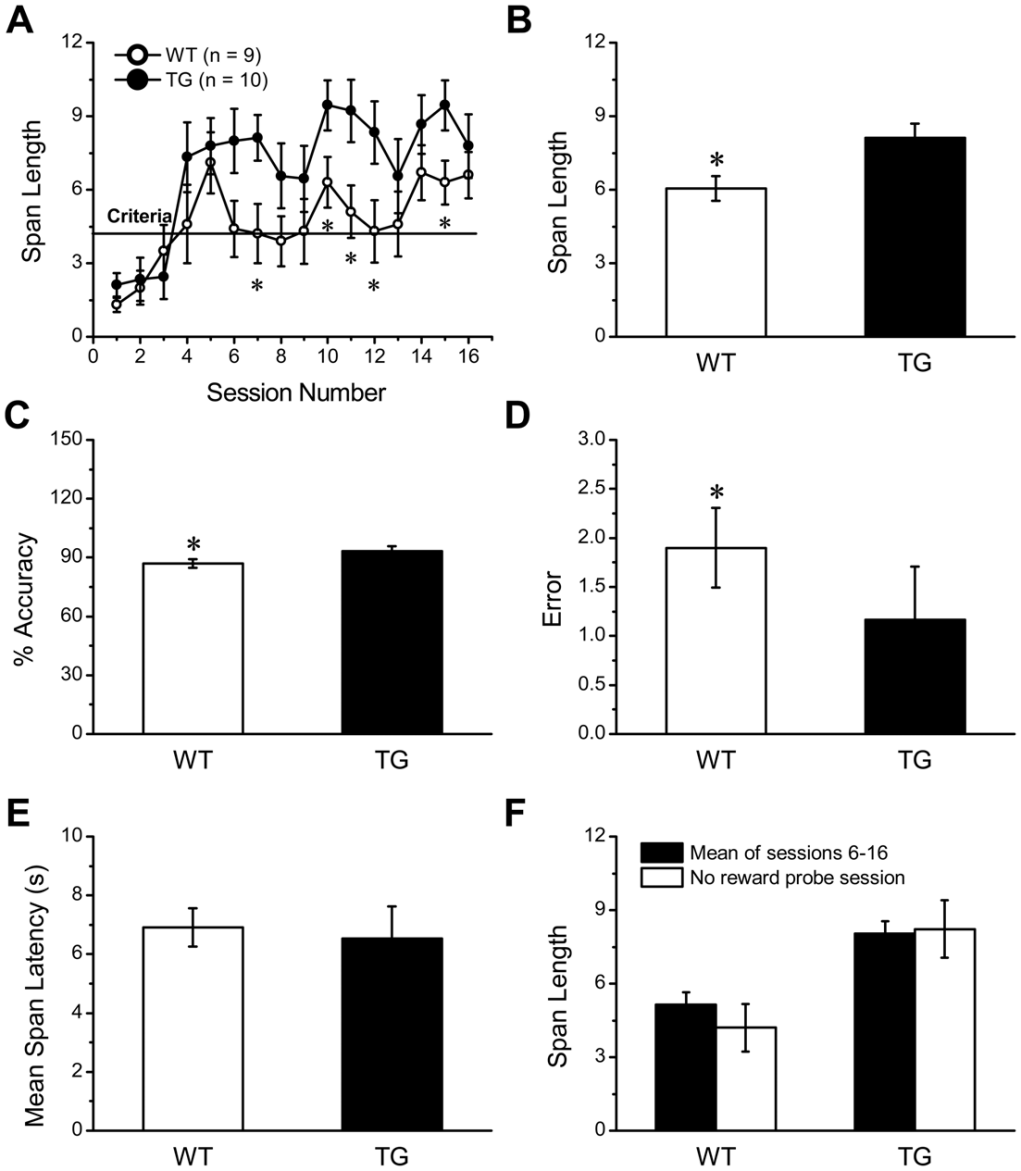
Enhancement of Non-spatial Working Memory in NR2B Transgenic Mice. A: Performance of the odor span task by transgenic mice and their control littlemate was compared over successive training 16 days. The Tg mice showed significantly improved performance on training days 7,10,11,12 (*p<0.05). B–E: the effect of genotype on stable performance (sessions 13–16) was assessed after Wt and Tg mice reached to a stable performance at sessions 13–16, a significant difference between the two groups was observed in span length (B), % accuracy (C) and error (D), but not in mean span length (E). F: In the no reward probe, the mean span length of each group was comparable with the mean span length of each group across across 11 sessions after the acquisition period (sessions 6–16). All values are mean ± SEM (*denotes p<0.05 when compared to Wt controls).

To further make sure that NR2B overexpression in prefrontal cortex certainly contributes to enhanced spatial working memory, the olfactory working memory of mice was assessed in the non-spatial cue dependent odor span task, which is hippocampus independent. The performance of the two groups in the odor span task across 16 sessions was compared using a two-way ANOVA. Significant main effects of both training day (F_(15,287)_ = 5.781, p<0.001, Figure 6A) and genotype (F_(1,287)_ = 29.965, p<0.001, Figure 6A) were observed, but there was no group-by-span length interaction (F_(15,287)_ = 0.86, p>0.05). Sidak-Holm post-hoc test analysis revealed that the performance of NR2B transgenic mice was significantly better than their wild type mice on sessions 7, 10, 11, 12, 15 (p<0.05). When both the Tg and Wt mice reached a span length ≥4.3, they performed significantly better than their performance on session 1 (p<0.05). This took 5 sessions for both Tg and Wt mice to reach this acquisition criterion (span length ≥4.3 for 2 consecutive sessions). Following attainment of acquisition criteria, all mice was continually trained to a stable level of performance with span length fluctuating within a maximum of 3 spans over 4 consecutive days. Since mice reached to a stable performance at sessions 13-16, the effect of genotype on stable performance was assessed by measuring simple 2-odor discrimination, span length, % accuracy, total errors and mean span latency of two groups across these sessions. Compared to the Wt mice, the Tg mice exhibited significantly higher span length (T = 456.5, p<0.01, Figure 6B), higher % accuracy (T = 405, p<0.001, Figure 6C) and fewer total errors (T = 418.5, p<0.001, Figure 6D). As there was no significant effect of genotype on mean span latency (F_(1,74)_ = 0.134, p>0.05 0.715, Figure 6E) and 2-odor discrimination (T = 702, p>0.05 0.343), the difference in span length between groups was not a consequence of the Tg mice being faster or more sensitive to odor.

In the no reward probe session, the mean span length of each group (Wt: 4.20±0.96; Tg: 8.22±1.18) did not differ from the mean span length of each group across 11 sessions after the acquisition period (sessions 6–16) (Wt: 5.15±0.48, p>0.05; Tg: 8.05±0.50, p>0.05, Figure 6F). Thus, it may be excluded that that mice did use the scent of the reward pellets to find the correct cup. The above data demonstrate that NR2B transgenic mice have better non-spatial working memory. Since odor span task is independent of hippocampus[17], enhanced non-spatial working memory in transgenic NR2B mice should correlate to overexpression of NR2B subunits in prefrontal cortex.

## Discussion

Most previous studies have focused on the role of NR2A and NR2B subunits in hippocampal long-term synaptic plasticity, LTP and LTD. However, there are still many conflict findings. Some pharmacological studies have shown that a selective NR2B subunit antagonist blocked LTD, but not LTP in the CA1 region of the hippocampus[26] and in the perirhinal cortex[27] while selective NR2A subunit antagonists had the opposite effects, blocking LTP but not LTD. Thus, it has been proposed that “NR2A triggering LTP/NR2B triggering LTD”.

However, other results from both genetic and pharmacological approaches were not consistent with the above proposition. For example, overexpression of NR2B enhanced LTP in the hippocampus[17] and activation of NR2B-containing NMDA receptors could generate LTP in mice lacking NR2A[28] or with impaired NR2A-mediated signaling[29]. These results suggest that NR2B subunit plays a key role in hippocampal LTP. In addition, three research groups (Stanford Group, UCSF group and MIT group) independently observed that the well-accepted selective antagonist of NR2B-containing NMDARs, ifenprodil, which clearly reduced NMDAR-mediated synaptic responses, did not affected LTD in the CA1 region of the hippocampus[30]. They proposed that activation of NR2B-containing NMDA receptors is not required for NMDA receptor-dependent LTD in hippocampus.

NR2A and NR2B subunits also predominate in the prefrontal cortex. However, to date, few studies have focused on the role of NMDA subunits in prefrontal cortex synaptic plasticity. In this study, we found overexpression of NR2B subunits did not affect the prefrontal basal synaptic transmission (I/O and paired-pulse facilitation) and LTD. However, the prefrontal cortex LTP in transgenic slices was significantly enhanced compared to wild type slices. Moreover, the enhanced LTP was blocked by APV, suggesting that the enhanced LTP is also mediated by postsynaptic NMDA receptors.

To further determine whether or not the robust enhancement of LTP is due to NR2B overexpression, we applied NR2A and (or) NR2B antagonist in electrophysiological experiments. When a selective NR2A subunit antagonist, NVP-AAM077, was added to ACSF, prefrontal cortex LTP of transgenic slices was still larger than that of Wt slices. Moreover, under treatment with a selective NR2B subunit antagonist, prefrontal cortex LTP of Tg slices was comparable with that of Wt slices. Taken together, this suggests that overexpression of NR2B subunit indeed contributes to the enhanced LTP, which is consistent with findings from hippocampus area of transgenic NR2B mice [17]. In addition, we found that antagonist of NR2A and (or) NR2B subunit reduced the prefrontal cortex LTP in both Tg and Wt slices. This result reconciled with the proposition that both NR2A and NR2B subunits were required for prefrontal cortex LTP [31].

Interestingly, Philpot [32] reported that overexpression of NR2B in forebrain did not alter LTP in visual cortex. One explanation for the diverse results is that expression of NR2B subunits were not increased in synaptosome of visual cortex in NR2B transgenic mice [32]. In contrast with the above result, our western blot data reveal that the synaptic expression of the NR2B protein was significantly increased in prefrontal cortex of NR2B transgenic mice. The increased expression of NR2B protein provides the molecular basis for the enhancement of NMDA-dependent LTP in the prefrontal cortex.

Working memory is a trial-unique-specific memory, which enables the temporary holding of information for the purposes of processing, playing a critical role in many cognitive tasks. Lesions restricted to PFC have been shown to impair performance on delayed-response tasks which reflect working memory ability [33]. Furthermore, antagonists of NMDA receptors impaired prefrontal cortex-dependent working memory, suggesting NMDAR have been implicated in working memory [11], [34] [35]. Based on all knowledge, we assume that overexpression of NR2B protein may enhance prefrontal-related working memory by up-regulating NMDA receptor function. Consistent with our speculation, NR2B transgenic mice exhibited super performance in comparision to Wt mice in T-maze and working-memory version of water maze tasks, suggesting NR2B overexpression can enhance spatial working memory.

Both hippocampus and prefrontal cortex play a role in spatial working memory [36], [37], [38], [39], moreover overexpression of NR2B gene is throughout the forebrain including hippocampus and prefrontal cortex in transgenic mice [17]. Therefore, it is difficult to conclude that the genetic enhancement of spatial working memory is due to NR2B overexpression in prefrontal cortex not in hippocampus. To further determine whether NR2B overexpresson in prefrontal cortex can enhance working memory, the odor span task, which is independent of hippocampus [21], was selected to evaluate the non-spatial cued working memory. NR2B transgenic mice also showed significantly enhanced non-spatial working memory as represented by an increased span length, higher percentage of accuracy and fewer errors. Therefore, it indicates that NR2B overexpression in prefrontal cortex may contribute to enhanced working memory. To establish the correlation of prefrontal NR2B overexpression with enhanced working memory, our future effort might be to overexpress NR2B subunit specifically in PFC or to perturb expression of NR2B specifically in PFC.

In summary, prefrontal over-expression of NR2B subunit not only facilitates prefrontal cortex long-term potentiation but also enhances prefrontal cortex-related working memory, suggesting NR2B subunit may also be a crucial switch for prefrontal cortex LTP and prefrontal cortex-related working memory.

Furthermore, a number of studies indicated that during the delay period of working memory tasks, neurons of the prefrontal cortex exhibited the elevated persistent firing activity [40], [41], [42], [43], [44]. If the persistent activity is disrupted by stimulation during the delay period, the animal is highly likely to make an error [45], [46], [47]. Thus, we hypothesize that during delay period of working memory task, the persistent neural activity of PFC in NR2B transgenic mice could be stronger than that of Wt mice. We will need to test our hypothesis in the future work.
